# Intra-Genomic Internal Transcribed Spacer Region Sequence Heterogeneity and Molecular Diagnosis in Clinical Microbiology

**DOI:** 10.3390/ijms161025067

**Published:** 2015-10-22

**Authors:** Ying Zhao, Chi-Ching Tsang, Meng Xiao, Jingwei Cheng, Yingchun Xu, Susanna K. P. Lau, Patrick C. Y. Woo

**Affiliations:** 1 Department of Clinical Laboratory, Peking Union Medical College Hospital, Chinese Academy of Medical Sciences, Beijing 100730, China; E-Mails: zhaoying28062806@163.com (Y.Z.); cjtcxiaomeng@aliyun.com (M.X.); zzujingwei@163.com (J.C.); xycpumch@139.com (Y.X.); 2 Department of Microbiology, The University of Hong Kong, Hong Kong; E-Mail: microbioct@connect.hku.hk; 3 Graduate School, Peking Union Medical College, Chinese Academy of Medical Sciences, Beijing, China; 4 State Key Laboratory of Emerging Infectious Diseases, The University of Hong Kong, Hong Kong; 5 Research Centre of Infection and Immunology, The University of Hong Kong, Hong Kong; 6 Carol Yu Centre for Infection, The University of Hong Kong, Hong Kong

**Keywords:** internal transcribed spacer region, sequencing, heterogeneity, yeast, molecular identification

## Abstract

Internal transcribed spacer region (ITS) sequencing is the most extensively used technology for accurate molecular identification of fungal pathogens in clinical microbiology laboratories. Intra-genomic ITS sequence heterogeneity, which makes fungal identification based on direct sequencing of PCR products difficult, has rarely been reported in pathogenic fungi. During the process of performing ITS sequencing on 71 yeast strains isolated from various clinical specimens, direct sequencing of the PCR products showed ambiguous sequences in six of them. After cloning the PCR products into plasmids for sequencing, interpretable sequencing electropherograms could be obtained. For each of the six isolates, 10–49 clones were selected for sequencing and two to seven intra-genomic ITS copies were detected. The identities of these six isolates were confirmed to be *Candida glabrata* (*n* = 2), *Pichia* (*Candida*) *norvegensis* (*n* = 2), *Candida tropicalis* (*n* = 1) and *Saccharomyces cerevisiae* (*n* = 1). Multiple sequence alignment revealed that one to four intra-genomic ITS polymorphic sites were present in the six isolates, and all these polymorphic sites were located in the ITS1 and/or ITS2 regions. We report and describe the first evidence of intra-genomic ITS sequence heterogeneity in four different pathogenic yeasts, which occurred exclusively in the ITS1 and ITS2 spacer regions for the six isolates in this study.

## 1. Introduction

Molecular identification of fungal pathogens in clinical microbiology laboratories most commonly involves polymerase chain reaction (PCR) amplification of genes and/or intergenic regions in the fungal genome and direct nucleotide sequencing of the purified PCR products. The most extensively used targets for such purpose are the internal transcribed spacer (ITS) region comprising the ITS1-5.8S-ITS2 nuclear ribosomal DNA (nrDNA) gene cluster and 25S nrDNA [1,2]. Since most fungi possess more than one nrDNA operon in their genomes [3,4], whether molecular characterization can be used to achieve fungal identification depends very much on the perfect intra-genomic sequence homogeneity in the multiple copies of the nrDNA operons of fungi.

Intra-genomic ITS sequence heterogeneity has rarely been described in pathogenic fungi. When present, such ITS sequence heterogeneity within a single genome makes fungal identification based on direct sequencing of the PCR product difficult, because multiple PCR products will result and double or multiple nucleotide peaks will be observed in the sequencing electropherograms. Recently, during the process of performing ITS sequencing on 71 isolates of yeasts recovered from various clinical specimens, direct sequencing of the PCR products showed ambiguous sequences in six of them (unpublished data). For these six isolates, which belong to four different fungal species, double or multiple peaks were frequently observed in the sequencing electropherograms. We hypothesize that intra-genomic ITS sequence heterogeneity is present in these six clinical isolates. In order to test for this hypothesis, the PCR products of the ITS of these six isolates were cloned and 10 to 49 clones from each isolate were selected for sequencing. In this study, we describe this phenomenon of ITS sequence heterogeneity within the genomes of four different pathogenic fungal species. The importance of intra-genomic ITS sequence heterogeneity on molecular identification and typing of fungal species in clinical microbiology laboratories is also discussed.

## 2. Results

### 2.1. Clinical Characteristics

The clinical characteristics of the five patients from whom the six yeast isolates were recovered are summarized in Table 1. Two patients were male and three were female, with a median age of 60 (range, 30 to 86). Two patients had type 2 diabetes mellitus, while one had cervical carcinoma and another one had chronic renal failure. All patients suffered from invasive yeast infections, including peritonitis, mediastinitis, as well as lung abscess and empyema, with the yeasts recovered from the corresponding clinical specimens. For the 66-year-old male patient with esophageal perforation and mediastinitis, yeasts were recovered from both the patient’s blood and pleural fluid, which were collected on separate days.

### 2.2. Direct PCR Product Sequencing

PCR of the ITS of the six patient isolates yielded DNA bands of about 900 bp (PUMY010 and PUMY011), 500 bp (PUMY020 and PUMY021), 550 bp (PUMY040) and 800 bp (PUMY065). Direct sequencing of the purified PCR products revealed that double or multiple peaks were present frequently in the sequencing electropherograms (Figure S1), and so the actual sequences of the ITS of the isolates could not be determined successfully. DNA extraction and direct PCR product sequencing were independently performed more than three times but the sequence ambiguity remained.

### 2.3. Sequencing of Cloned PCR Products and Phylogenetic Analyses

**Figure 1:**
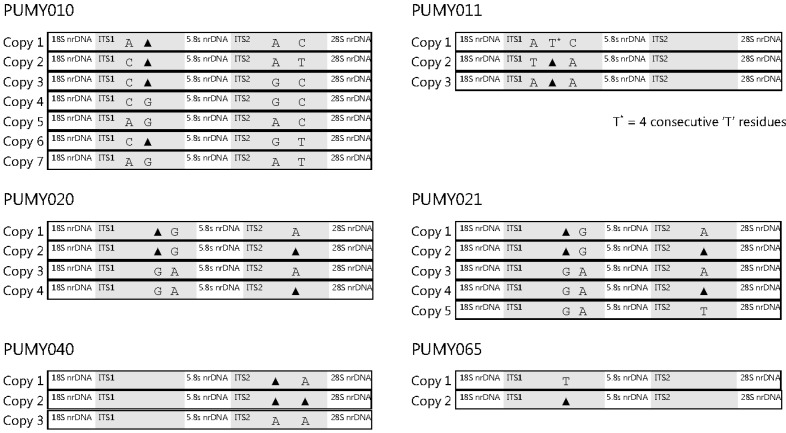
Schematic diagrams showing the multiple alignments of ITS sequences of the six yeast isolates. The internal transcribed spacer regions ITS1 and ITS2 are highlighted in gray. Intra-genomic polymorphisms are represented by the corresponding nucleotides or the symbol “▲” (meaning deletion) at the respective sites. The diagrams are not drawn to scale.

**Figure 2:**
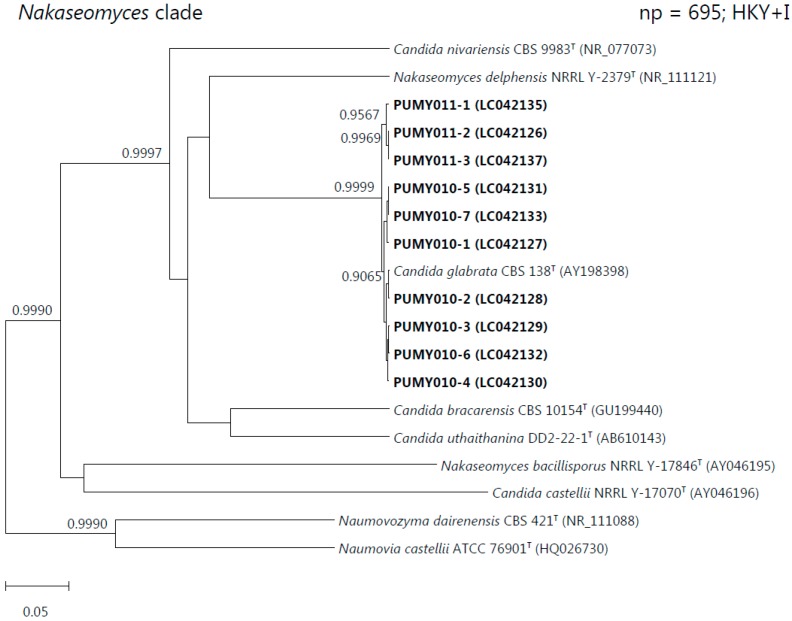

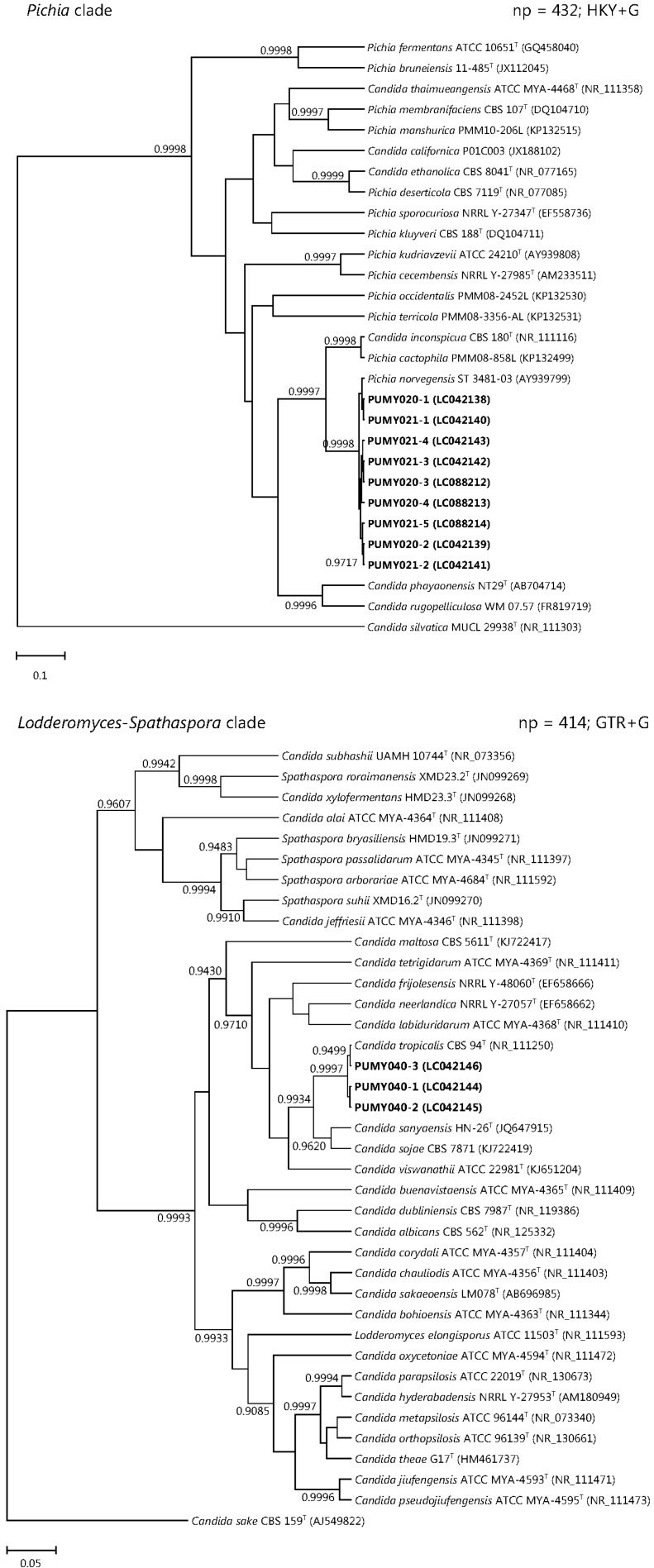

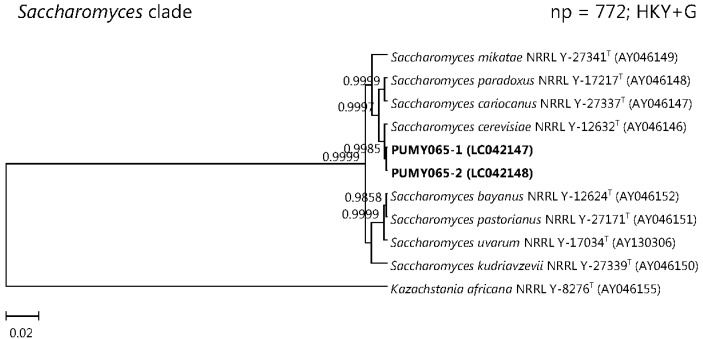
Phylogenetic trees showing the relationship of the six yeast isolates to other members of the *Nakaseomyces*, *Pichia*, *Lodderomyces-Spathaspora*, or *Saccharomyces* clades. The trees were inferred from ITS sequence data by the Bayesian inference method and the numbers of nucleotide positions (np) of the trimmed, aligned sequences included and the substitution models employed (HKY = Hasegawa-Kishino-Yano model; GTR = general time reversible model; G = γ-distributed rate variation; and I = estimated proportion of invariable sites) for phylogenetic analyses are shown at the top right corner of each tree, and the trees were rooted using *Candida* *castellii* NRRL-17070^T^, *C. silvatica* MUCL 29938^T^, *C. sake* CBS 159^T^, and *Kazachstania africana* NRRL Y-8276^T^, respectively. The scale bars indicate the estimated numbers of substitutions per base. Numbers at nodes indicate levels of posterior probability support based on the Bayesian analyses of the data sets, and posterior probability values lower than 0.9 are not shown. All accession numbers (in parentheses) are given as cited in the DDBJ/ENA/GenBank databases. The clinical isolates reported in this study are highlighted in bold type.

After cloning the PCR products into plasmids for sequencing, interpretable sequencing electropherograms could be obtained (Figure S1). Out of the 17, 12, 45, 49, 10 and 15 clones selected for sequencing for the isolates PUMY010, PUMY011, PUMY020, PUMY021, PUMY040 and PUMY065, respectively, two to seven intra-genomic ITS copies were observed (Table 1 and Figure S1). Multiple sequence alignment revealed that one to four intra-genomic ITS polymorphic sites were present in the six isolates, and all these polymorphic sites were located in the ITS1 and/or ITS2 regions (Table 1, Figure 1, Figures S1 and S2). For PUMY010, the four polymorphic sites present in the seven intra-genomic ITS copies involved transition (A ↔ G and T ↔ C) and transversion (A ↔ C) substitutions as well as an insertion/deletion, and these intra-genomic ITS copies possessed 99.05%–99.41% sequence identities to the ITS of *Candida glabrata* CBS 138^T^; for PUMY011, the three polymorphic sites present in the three intra-genomic ITS copies involved transversion (A ↔ T and A ↔ C) substitutions as well as an insertion/deletion, and these intra-genomic ITS copies possessed 98.82%–99.29% sequence identities to the ITS of *C. glabrata* CBS 138^T^; for PUMY020, the three polymorphic sites present in the four intra-genomic ITS copies involved a transition (A ↔ G) substitution and insertions/deletions, and these intra-genomic ITS copies possessed 98.68%–99.12% sequence identities to the ITS of *Pichia* (*Candida*) *norvegensis* ST 3481-03; for PUMY021, the three polymorphic sites present in the five intra-genomic ITS copies involved transition (A ↔ G) and transversion (A ↔ T) substitutions as well as insertions/deletions, and these intra-genomic ITS copies possessed 98.68%–99.12% sequence identities to the ITS of *P. norvegensis* ST 3481-03; for PUMY040, the two polymorphic sites present in the three intra-genomic ITS copies involved only insertions/deletions and these intra-genomic ITS copies possessed 99.59%–100% sequence identities to the ITS of *C. tropicalis* CBS 94^T^; while for PUMY065, the single polymorphic site present in the two intra-genomic ITS copies involved only an insertion/deletion and these intra-genomic ITS copies possessed 99.73%–99.87% sequence identities to the ITS of *Saccharomyces cerevisiae* NRRL Y-12632^T^. Phylogenetic analyses based on all the intra-genomic ITS copies of each yeast isolate showed unambiguously that isolates PUMY010 and PUMY011 were clustered with *C. glabrata* CBS 138^T^; isolates PUMY020 and PUMY021 were clustered with *P. norvegensis* ST 3481-03; isolate PUMY040 was clustered with *C. tropicalis* CBS 94^T^; and isolate PUMY065 was clustered with *S. cerevisiae* NRRL Y-12632^T^ (Figure 2).

## 3. Discussion

We report and describe the first evidence of intra-genomic ITS sequence heterogeneity in a variety of yeast species recovered from clinical specimens. So far, most cases of intra-genomic ITS sequence heterogeneity were reported in environmental fungi of several fungal phyla, including Ascomycota [5,6,7,8,9], Basidiomycota [6,10,11,12,13], Glomeromycota [14,15,16,17,18,19,20], Microsporidia [21,22] and the subphylum Mucoromycotina [23]. Most of these fungi are molds, whereas only three yeasts species [6] and one dimorphic fungus [8] have been found to possess intra-genomic ITS sequence heterogeneity. On the other hand, this phenomenon has rarely been reported in fungal pathogens identified in clinical microbiology laboratories. In our previous study, we documented the presence of intra-genomic ITS sequence heterogeneity in four of the 28 *Rhizopus microsporus* isolates recovered during an outbreak investigation of gastrointestinal mucormycosis [23]. In the present study, this phenomenon could also be observed in six of the 71 yeast isolates subject to ITS sequencing. These six isolates, of four different fungal species, were isolated from five patients who suffered from severe fungal infections due to the corresponding yeasts (Table 1). As one of the major reasons for intra-genomic ITS sequence heterogeneity is the result of DNA insertion/deletion, minute differences in the sizes of the PCR products would be generated during amplification of the ITS region. This may give rise to thicker bands observed in agarose gel electrophoresis if the nucleotide fragments inserted/deleted is long enough, as we observed in our previous study [11]. In the present study, this “thicker band” phenomenon was not obvious for all the six isolates as the different intra-genomic ITS copies observed were only due to substitutions and/or very short insertions/deletions involving only one to four nucleotides. More importantly, multiple peaks were always observed in the sequencing electropherograms if the PCR products were sequenced directly since two or more types of PCR products were simultaneously sequenced (Figure S1). In this study, cloning and subsequent sequencing of the ITS PCR products of the six yeast isolates showed that for each of the six isolates, more than one type of ITS sequences were observed (Figure 1, Figures S1 and S2). This confirmed intra-genomic ITS sequence heterogeneity in all six yeast isolates. In addition, all the four ITS copies of the isolate PUMY020 could also be found in the isolate PUMY021 (copies 1–4). Although PUMY021 possessed an additional fifth ITS copy which could not be observed in PUMY020, the electropherogram of PUMY020 obtained by direct PCR product sequencing showed that the presence of such an ITS copy (with ten “T” residues followed by two “A” residues at the ITS2 polymorphic site) in the genome of PUMY020 is possible (Figure S1). Given the recovery rate of this ITS copy in PUMY021 was just one in 49 clones, it is possible that the clones selected for sequencing were not sufficient in number to reveal this ITS copy in PUMY020. As a result, it is reasonable to believe that the isolates PUMY020 and PUMY021, which were isolated from the same patient from different clinical specimens collected on different dates, are actually the same.

Concerted evolutionary mechanisms are employed to maintain sequence uniformity among the many different copies of the nrDNA operon within a fungal genome. However, when error occurs, intra-genomic nrDNA sequence variation would result and this gives rise to nrDNA sequence heterogeneity within a single genome. As for the positions of intra-genomic ITS sequence variation, they occurred only in ITS1 and/or ITS2, but not in the 5.8S nrDNA region, for all the six yeast isolates in this study (Figures 1 and S2). In fungi which possess intra-genomic ITS sequence heterogeneity, the sites of nucleotide difference are also much more often present in ITS1 and/or ITS2, and intra-genomic 5.8S nrDNA variation is uncommonly observed [5,7,8,9,10,11,12,17,18,19,20,21,22,23]. This phenomenon may likely be explained by the fact that the 5.8S nrRNA, after maturation and together with the mature 25S and 5S rRNAs, forms the large ribosomal subunit, which is essential to the organism for protein translation. Given the importance of the function of the 5.8S nrRNA and its short length of about 160 nucleotides, it is not surprising to observe minimal sequence variation in this gene. On the other hand, although ITS1 and ITS2 are also transcribed together with the 18S, 5.8S and 25S nrDNA to give the 45S rRNA precursor, upon maturation these two spacers are spliced and removed and they are not involved in the functioning of the ribosome. Therefore, the two spacers ITS1 and ITS2 are under fewer evolutionary constraints than the 5.8S nrDNA and thus more sequence variations could be observed.

**Table 1:** Characteristics of the six yeast strains in this study.

Patient	Sex/Age _a_	Underlying Diseases _b_	Clinical Diagnosis	Clinical Specimens	Strains _c_	Number of Intra-Genomic ITS Copies _d_	Number of Intra-Genomic ITS Polymorphic Sites _d_	Location of Intra-Genomic ITS Polymorphic Sites _d_	Final Identification
1	F/30	Carcinoma of cervix, post-total abdominal hysterectomy and bilateral salpingo-oophorectomy and radiotherapy	Suppurative peritonitis	Peritoneal fluid	PUMY010	7	4	ITS1 and ITS2	*Candida glabrata*
2	F/86	N/A	Intestinal obstruction	Intraperitoneal drainage fluid	PUMY011	3	3	ITS1	*Candida glabrata*
3	M/66	Type 2 diabetes mellitus	Esophageal perforation and mediastinitis	Pleural fluid	PUMY020	4	3	ITS1 and ITS2	*Pichia* (*Candida*) *norvegensis*
				Blood	PUMY021	5	3	ITS1 and ITS2	*Pichia* (*Candida*) *norvegensis*
4	M/60	Chronic renal failure	Peritonitis	Peritoneal fluid	PUMY040	3	2	ITS2	*Candida tropicalis*
5	F/58	Type 2 diabetes mellitus	Lung abscess and empyema	Empyema pus	PUMY065	2	1	ITS1	*Saccharomyces cerevisiae*

^a^ F, female; M, male; ^b^ N/A, not available; ^c^ Strain PUMY021 was isolated two days after the isolation of strain PUMY20 from the same patient; ^d^ ITS, internal transcribed spacer.

Intra-genomic ITS sequence heterogeneity poses difficulties in both the identification and typing of the corresponding fungal strains. Rapid and accurate identification of fungal pathogens is particularly important to clinical microbiology laboratories because it is essential to the prescription of the appropriate antifungal treatment to the patients. In the presence of intra-genomic ITS sequence heterogeneity, the direct sequencing results of the PCR products may be uninterpretable and it takes much more time to clone the PCR products for subsequent sequencing to obtain interpretable sequences corresponding to the different intra-genomic ITS copies. The additional experimental steps may require skilled personnel and the lengthy procedures may hinder the prescription of the correct treatment regimens in a timely manner. As for strain- or geno-typing of the fungal pathogen, which are important to the study of infection epidemiology, the different intra-genomic ITS copies may complicate the typing analysis. For example, in the *Nakaseomyces* clade phylogenetic tree in this study, the seven ITS sequence types of the isolate PUMY010 formed three distinct clusters and these three separated clusters may represent three individual strain types (Figure 2). Such phenomenon could also be observed in other yeast isolates characterized in this study (Figure 2). This makes it difficult to conclude to which strain types these isolates actually belong and hence hinders epidemiological study. Therefore, when double or multiple peaks of strong signal intensities are observed in the sequencing electropherograms of ITS PCR products, reference laboratories proficient in molecular technology should be consulted for further identification and typing of such fungal strains.

## 4. Materials and Methods

### 4.1. Patients and Strains

The six strains used in this study were isolated from clinical specimens of five patients hospitalized in Peking Union Medical College Hospital (PUMCH), Beijing, China, during a period of 10 months (May 2009 to February 2010) (Table 1). The medical records of the patients were retrospectively reviewed and their demographic information and clinical statuses (including sex, age, underlying diseases, clinical diagnosis, and type of specimens from which the yeast strains were isolated) were collected. This study was approved by the Human Research Ethics Committee of PUMCH (Number S-263, dated 28 October 2009), where the study protocols were performed in accordance with the approved guidelines and written informed consents were obtained.

### 4.2. DNA Extraction, PCR and Direct PCR Product Sequencing

Fungal genomic DNA was extracted from a single clone of each yeast isolate. Briefly, for each yeast isolate, cells from a single colony were subcultured on Sabouraud dextrose agar (SDA) (Oxoid, Hampshire, UK) supplemented with chloramphenicol (50 µg/mL) (Sigma-Aldrich, St. Louis, MO, USA). After 24–48 h of incubation at 25 °C the fresh cells were harvested with a sterile cotton swab and suspended in 1 mL of autoclaved distilled water with glass beads (212–300 µm in diameter) (Sigma-Aldrich). The cells were then disrupted using TissueLyser II (QIAGEN, Hilden, Germany) at a frequency of 30 Hz for 1 min. Genomic DNA from the homogenized cell suspension was then purified using the QIAquick PCR Purification Kit (QIAGEN) according to the manufacturer’s protocol. Subsequent PCR amplification and DNA sequencing of the ITS region for the six yeast isolates were performed according to our previous publication [24] using the primer pair ITS1/ITS4 [25]. The sequencing electropherograms obtained were viewed using Chromas Lite 2.1.1 (Technelysium, South Brisbane, Australia). For each yeast isolate, PCR amplification was performed at least twice to ensure that the sequence ambiguity was not due to DNA elongation error of the polymerase.

### 4.3. Cloning and Sequencing

Freshly-prepared gel-purified PCR products were cloned into the pCRII-TOPO vector using the TOPO TA Cloning Kit (Invitrogen, Carlsbad, CA, USA) according to the manufacturer’s protocol. The TA-ligated plasmids were then transformed into *Escherichia coli* DH5α (TaKaRa Bio, Kusatsu, Japan) by electroporation. The electroporated cells were then grown on LB agar, Lennox (Difco, BD Diagnostic Systems, Sparks, MD, USA) with kanamycin (50 µg/mL) (Sigma-Aldrich) for the selection of positive transformants, as well as isopropyl β-d-1-thiogalactopyranoside (IPTG) (40 µg/mL) (Sigma-Aldrich) and 5-bromo-4-chloro-3-indolyl-beta-d-galactopyranoside (X-gal) (100 µg/mL) (Promega, Madison, WI, USA) for blue-white screening. White colonies were then selected and grown in LB broth, Lennox (Difco) with kanamycin (50 µg/mL) overnight and the plasmids in the bacterial cells were extracted using the QIAprep Spin Miniprep Kit (QIAGEN) according to the manufacturer’s protocol. The purified plasmids were then sequenced using the PCR primers directly. The sequencing electropherograms obtained were viewed using Chromas Lite 2.1.1.

### 4.4. Comparative Sequence Identity Analyses and Phylogenetic Analyses

The sequences obtained from the cloned PCR products were comparatively analyzed by pairwise alignment, with the optimal GLOBAL alignment parameters, using BioEdit 7.2.0 [26]. The sequences of the PCR products were also compared with sequences of closely related species from GenBank by multiple sequence alignment using MUSCLE 3.8 [27] and were then end-trimmed. Poorly aligned or divergent regions of the aligned, end-trimmed DNA sequences were removed using Gblocks 0.91b [28,29] with relaxed parameters. Tests for substitution models were performed using MEGA 6.06 [30] and phylogenetic tree construction, by the Bayesian inference method, was performed using BEAST 1.8.0 [31]. For each analysis, ten million generations were run with trees sampled every 1,000th generation to yield 10,000 trees. The trees were then summarized as a single consensus tree for each clade using TreeAnnotator 1.8.0 and viewed using FigTree 1.4.0.

### 4.5. Nucleotide Sequence Accession Number

The ITS sequences of the six yeast isolates have been deposited in the DDBJ/ENA/GenBank databases with the accession numbers LC042127-LC042133, LC042135-LC042148, and LC088212-LC088214.

## Supplementary Materials

Supplementary materials can be found at http://www.mdpi.com/1422-0067/16/10/25067/s1.
