# Supplementary material for: Intra-Genomic Internal Transcribed Spacer Region Sequence Heterogeneity and Molecular Diagnosis in Clinical Microbiology
Source: Int J Mol Sci. 2015 Oct 22;16(10):25067–79. doi: 10.3390/ijms161025067 (PMC4632790; doi:10.3390/ijms161025067)
Supplement: Supplementary file 1 [file ijms-16-25067-s001.pdf]

Supplementary Information

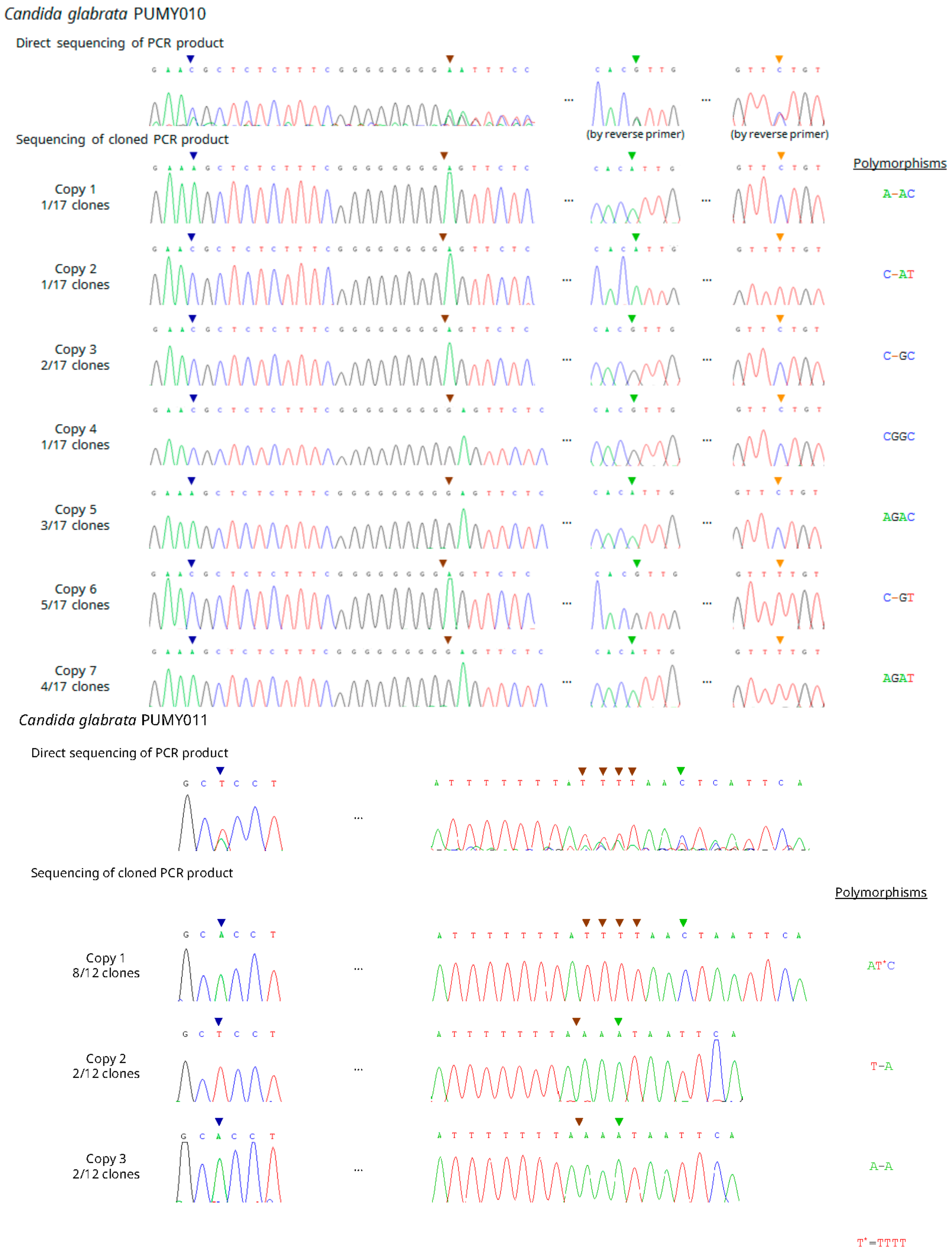

Figure S1. *Cont.*

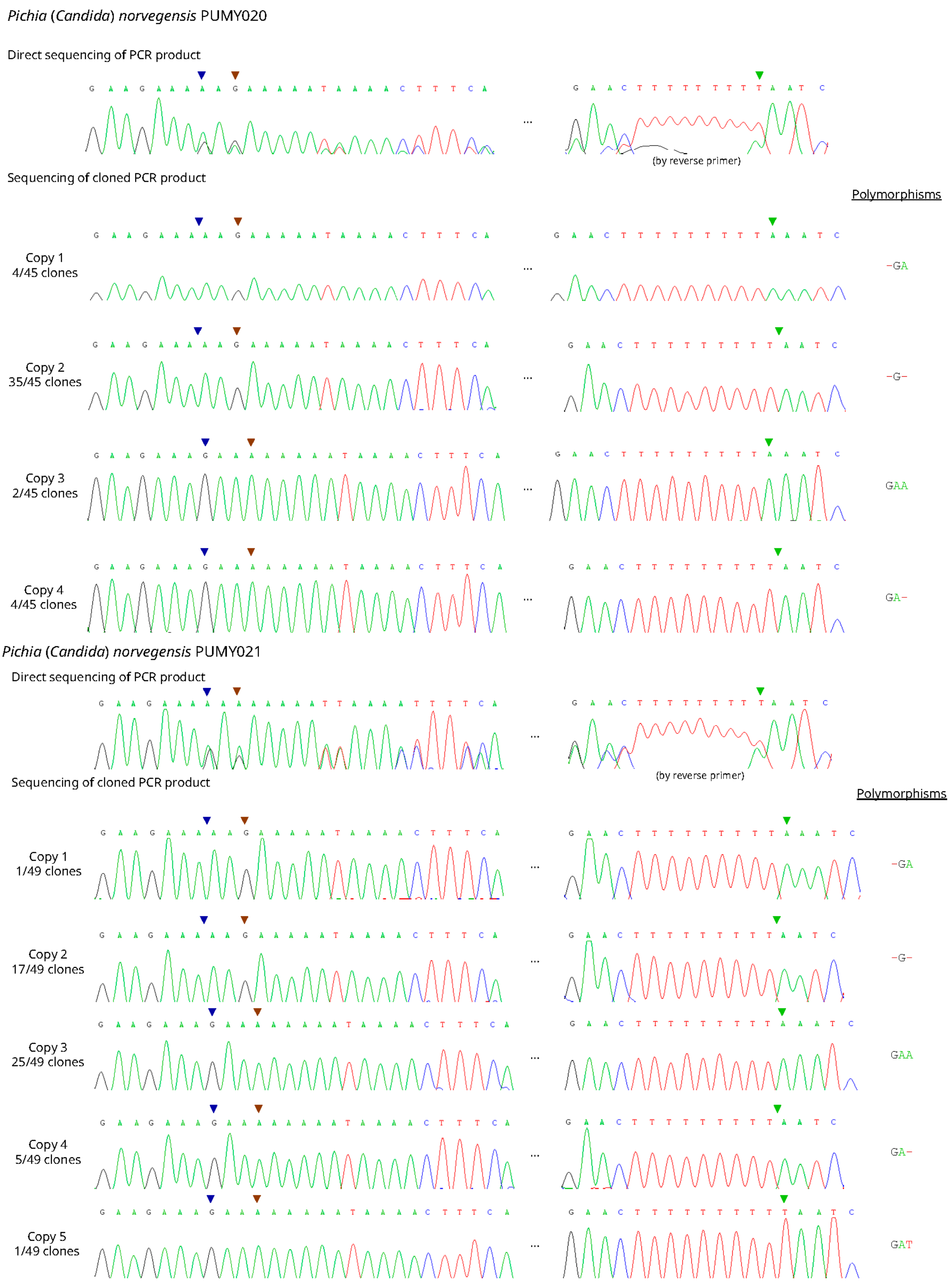

Figure S1. *Cont.*

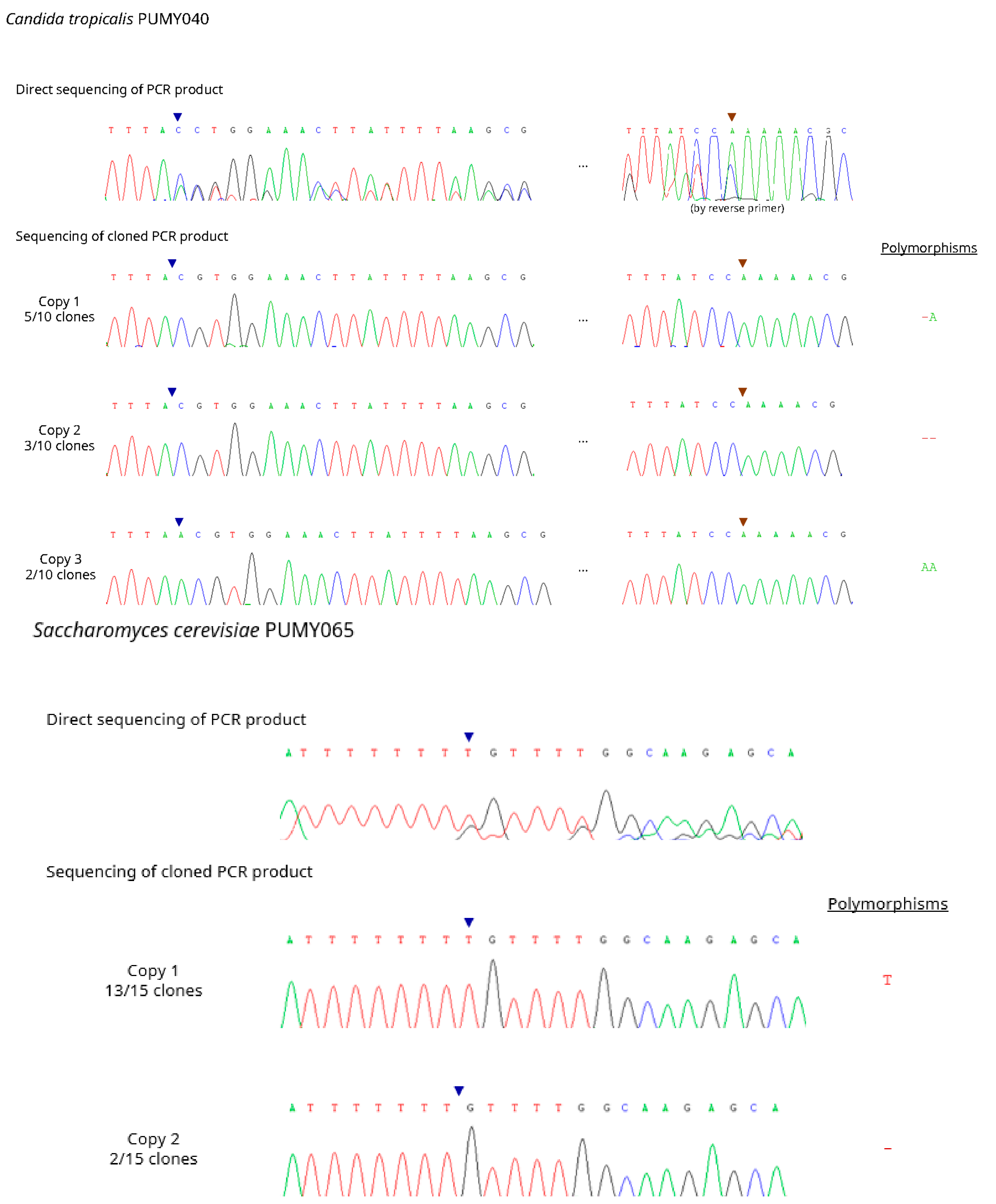

Figure S1. Sequencing electropherograms of the directly purified and cloned PCR products for the six yeast strains. The intra-genomic polymorphic sites are indicated by inverted arrowheads in different colors, and each color refers to a single polymorphic site.

```
              18S nrDNA   ITS1
PUMY010-1     AAGGATCATT AAAGAAATTT AATTGATTTG TCTGAGCTCG GAGAGAGACA    50
PUMY010-2     AAGGATCATT AAAGAAATTT AATTGATTTG TCTGAGCTCG GAGAGAGACA    50
PUMY010-3     AAGGATCATT AAAGAAATTT AATTGATTTG TCTGAGCTCG GAGAGAGACA    50
PUMY010-4     AAGGATCATT AAAGAAATTT AATTGATTTG TCTGAGCTCG GAGAGAGACA    50
PUMY010-5     AAGGATCATT AAAGAAATTT AATTGATTTG TCTGAGCTCG GAGAGAGACA    50
PUMY010-6     AAGGATCATT AAAGAAATTT AATTGATTTG TCTGAGCTCG GAGAGAGACA    50
PUMY010-7     AAGGATCATT AAAGAAATTT AATTGATTTG TCTGAGCTCG GAGAGAGACA    50

PUMY010-1     TCTCTGGGGA GGACCAGTGT AGACACTCAG GAGGTTCCTA AAATATTTTC    100
PUMY010-2     TCTCTGGGGA GGACCAGTGT AGACACTCAG GAGGTTCCTA AAATATTTTC    100
PUMY010-3     TCTCTGGGGA GGACCAGTGT AGACACTCAG GAGGTTCCTA AAATATTTTC    100
PUMY010-4     TCTCTGGGGA GGACCAGTGT AGACACTCAG GAGGTTCCTA AAATATTTTC    100
PUMY010-5     TCTCTGGGGA GGACCAGTGT AGACACTCAG GAGGTTCCTA AAATATTTTC    100
PUMY010-6     TCTCTGGGGA GGACCAGTGT AGACACTCAG GAGGTTCCTA AAATATTTTC    100
PUMY010-7     TCTCTGGGGA GGACCAGTGT AGACACTCAG GAGGTTCCTA AAATATTTTC    100

PUMY010-1     TCTGCTGTGA ATGCCATTTC TCCTGCCTGC GCTTAAGTGC GCGGTTGGTG    150
PUMY010-2     TCTGCTGTGA ATGCCATTTC TCCTGCCTGC GCTTAAGTGC GCGGTTGGTG    150
PUMY010-3     TCTGCTGTGA ATGCCATTTC TCCTGCCTGC GCTTAAGTGC GCGGTTGGTG    150
PUMY010-4     TCTGCTGTGA ATGCCATTTC TCCTGCCTGC GCTTAAGTGC GCGGTTGGTG    150
PUMY010-5     TCTGCTGTGA ATGCCATTTC TCCTGCCTGC GCTTAAGTGC GCGGTTGGTG    150
PUMY010-6     TCTGCTGTGA ATGCCATTTC TCCTGCCTGC GCTTAAGTGC GCGGTTGGTG    150
PUMY010-7     TCTGCTGTGA ATGCCATTTC TCCTGCCTGC GCTTAAGTGC GCGGTTGGTG    150

PUMY010-1     GGTGTTCTGC AGTGGGGGGA GGGAGCCGAC AAAGACCTGG GAGTGTGCGT    200
PUMY010-2     GGTGTTCTGC AGTGGGGGGA GGGAGCCGAC AAAGACCTGG GAGTGTGCGT    200
PUMY010-3     GGTGTTCTGC AGTGGGGGGA GGGAGCCGAC AAAGACCTGG GAGTGTGCGT    200
PUMY010-4     GGTGTTCTGC AGTGGGGGGA GGGAGCCGAC AAAGACCTGG GAGTGTGCGT    200
PUMY010-5     GGTGTTCTGC AGTGGGGGGA GGGAGCCGAC AAAGACCTGG GAGTGTGCGT    200
PUMY010-6     GGTGTTCTGC AGTGGGGGGA GGGAGCCGAC AAAGACCTGG GAGTGTGCGT    200
PUMY010-7     GGTGTTCTGC AGTGGGGGGA GGGAGCCGAC AAAGACCTGG GAGTGTGCGT    200

PUMY010-1     GGATCTCTCT ATTCCAAAGG AGGTGTTTTA TCACACGACT CGACACTTTC    250
PUMY010-2     GGATCTCTCT ATTCCAAAGG AGGTGTTTTA TCACACGACT CGACACTTTC    250
PUMY010-3     GGATCTCTCT ATTCCAAAGG AGGTGTTTTA TCACACGACT CGACACTTTC    250
PUMY010-4     GGATCTCTCT ATTCCAAAGG AGGTGTTTTA TCACACGACT CGACACTTTC    250
PUMY010-5     GGATCTCTCT ATTCCAAAGG AGGTGTTTTA TCACACGACT CGACACTTTC    250
PUMY010-6     GGATCTCTCT ATTCCAAAGG AGGTGTTTTA TCACACGACT CGACACTTTC    250
PUMY010-7     GGATCTCTCT ATTCCAAAGG AGGTGTTTTA TCACACGACT CGACACTTTC    250

PUMY010-1     TAATTACTAC ACACAGTGGA GTTTACTTTA CTACTATTCT TTTGTTCGTT    300
PUMY010-2     TAATTACTAC ACACAGTGGA GTTTACTTTA CTACTATTCT TTTGTTCGTT    300
PUMY010-3     TAATTACTAC ACACAGTGGA GTTTACTTTA CTACTATTCT TTTGTTCGTT    300
PUMY010-4     TAATTACTAC ACACAGTGGA GTTTACTTTA CTACTATTCT TTTGTTCGTT    300
PUMY010-5     TAATTACTAC ACACAGTGGA GTTTACTTTA CTACTATTCT TTTGTTCGTT    300
PUMY010-6     TAATTACTAC ACACAGTGGA GTTTACTTTA CTACTATTCT TTTGTTCGTT    300
PUMY010-7     TAATTACTAC ACACAGTGGA GTTTACTTTA CTACTATTCT TTTGTTCGTT    300
                     •            •
PUMY010-1     GGGGGAAAGC TCTCTTTC-G GGGGGGGAGT TCTCCCAGTG GATGCAAACA    349
PUMY010-2     GGGGGAACGC TCTCTTTC-G GGGGGGGAGT TCTCCCAGTG GATGCAAACA    349
PUMY010-3     GGGGGAACGC TCTCTTTC-G GGGGGGGAGT TCTCCCAGTG GATGCAAACA    349
PUMY010-4     GGGGGAACGC TCTCTTTCGG GGGGGGGAGT TCTCCCAGTG GATGCAAACA    350
PUMY010-5     GGGGGAAAGC TCTCTTTCGG GGGGGGGAGT TCTCCCAGTG GATGCAAACA    350
PUMY010-6     GGGGGAACGC TCTCTTTC-G GGGGGGGAGT TCTCCCAGTG GATGCAAACA    349
PUMY010-7     GGGGGAAAGC TCTCTTTCGG GGGGGGGAGT TCTCCCAGTG GATGCAAACA    350

PUMY010-1     CAAACAAATA TTTTTTTAAA CTAATTCAGT CAACACAAGA TTTCTTTTAG    399
PUMY010-2     CAAACAAATA TTTTTTTAAA CTAATTCAGT CAACACAAGA TTTCTTTTAG    399
PUMY010-3     CAAACAAATA TTTTTTTAAA CTAATTCAGT CAACACAAGA TTTCTTTTAG    399
PUMY010-4     CAAACAAATA TTTTTTTAAA CTAATTCAGT CAACACAAGA TTTCTTTTAG    400
PUMY010-5     CAAACAAATA TTTTTTTAAA CTAATTCAGT CAACACAAGA TTTCTTTTAG    400
PUMY010-6     CAAACAAATA TTTTTTTAAA CTAATTCAGT CAACACAAGA TTTCTTTTAG    399
PUMY010-7     CAAACAAATA TTTTTTTAAA CTAATTCAGT CAACACAAGA TTTCTTTTAG    400
                               5.8S nrDNA
PUMY010-1     TAGAAAACAA CTTCAAAACT TTCAACAATG GATCTCTTGG TTCTCGCATC    449
PUMY010-2     TAGAAAACAA CTTCAAAACT TTCAACAATG GATCTCTTGG TTCTCGCATC    449
PUMY010-3     TAGAAAACAA CTTCAAAACT TTCAACAATG GATCTCTTGG TTCTCGCATC    449
PUMY010-4     TAGAAAACAA CTTCAAAACT TTCAACAATG GATCTCTTGG TTCTCGCATC    450
PUMY010-5     TAGAAAACAA CTTCAAAACT TTCAACAATG GATCTCTTGG TTCTCGCATC    450
PUMY010-6     TAGAAAACAA CTTCAAAACT TTCAACAATG GATCTCTTGG TTCTCGCATC    449
PUMY010-7     TAGAAAACAA CTTCAAAACT TTCAACAATG GATCTCTTGG TTCTCGCATC    450

PUMY010-1     GATGAAGAAC GCAGCGAAAT GCGATACGTA ATGTGAATTG CAGAATTCCG    499
PUMY010-2     GATGAAGAAC GCAGCGAAAT GCGATACGTA ATGTGAATTG CAGAATTCCG    499
PUMY010-3     GATGAAGAAC GCAGCGAAAT GCGATACGTA ATGTGAATTG CAGAATTCCG    499
PUMY010-4     GATGAAGAAC GCAGCGAAAT GCGATACGTA ATGTGAATTG CAGAATTCCG    500
PUMY010-5     GATGAAGAAC GCAGCGAAAT GCGATACGTA ATGTGAATTG CAGAATTCCG    500
PUMY010-6     GATGAAGAAC GCAGCGAAAT GCGATACGTA ATGTGAATTG CAGAATTCCG    499
PUMY010-7     GATGAAGAAC GCAGCGAAAT GCGATACGTA ATGTGAATTG CAGAATTCCG    500

PUMY010-1     TGAATCATCG AATCTTTGAA CGCACATTGC GCCCTCTGGT ATTCCGGGGG    549
PUMY010-2     TGAATCATCG AATCTTTGAA CGCACATTGC GCCCTCTGGT ATTCCGGGGG    549
PUMY010-3     TGAATCATCG AATCTTTGAA CGCACATTGC GCCCTCTGGT ATTCCGGGGG    549
PUMY010-4     TGAATCATCG AATCTTTGAA CGCACATTGC GCCCTCTGGT ATTCCGGGGG    550
PUMY010-5     TGAATCATCG AATCTTTGAA CGCACATTGC GCCCTCTGGT ATTCCGGGGG    550
PUMY010-6     TGAATCATCG AATCTTTGAA CGCACATTGC GCCCTCTGGT ATTCCGGGGG    549
PUMY010-7     TGAATCATCG AATCTTTGAA CGCACATTGC GCCCTCTGGT ATTCCGGGGG    550
```

Figure S2. *Cont.*

```
                                    ITS2         ●
PUMY010-1    GCATGCCTGT TTGAGCGTCA TTTCCTTCTC AAACACATTG TGTTTGGTAG    599
PUMY010-2    GCATGCCTGT TTGAGCGTCA TTTCCTTCTC AAACACATTG TGTTTGGTAG    599
PUMY010-3    GCATGCCTGT TTGAGCGTCA TTTCCTTCTC AAACACGTTG TGTTTGGTAG    599
PUMY010-4    GCATGCCTGT TTGAGCGTCA TTTCCTTCTC AAACACGTTG TGTTTGGTAG    600
PUMY010-5    GCATGCCTGT TTGAGCGTCA TTTCCTTCTC AAACACATTG TGTTTGGTAG    600
PUMY010-6    GCATGCCTGT TTGAGCGTCA TTTCCTTCTC AAACACGTTG TGTTTGGTAG    599
PUMY010-7    GCATGCCTGT TTGAGCGTCA TTTCCTTCTC AAACACATTG TGTTTGGTAG    600

PUMY010-1    TGAGTGATAC TCTCGTTTTT GAGTTAACTT GAAATTGTAG GCCATATCAG    649
PUMY010-2    TGAGTGATAC TCTCGTTTTT GAGTTAACTT GAAATTGTAG GCCATATCAG    649
PUMY010-3    TGAGTGATAC TCTCGTTTTT GAGTTAACTT GAAATTGTAG GCCATATCAG    649
PUMY010-4    TGAGTGATAC TCTCGTTTTT GAGTTAACTT GAAATTGTAG GCCATATCAG    650
PUMY010-5    TGAGTGATAC TCTCGTTTTT GAGTTAACTT GAAATTGTAG GCCATATCAG    650
PUMY010-6    TGAGTGATAC TCTCGTTTTT GAGTTAACTT GAAATTGTAG GCCATATCAG    649
PUMY010-7    TGAGTGATAC TCTCGTTTTT GAGTTAACTT GAAATTGTAG GCCATATCAG    650

PUMY010-1    TATGTGGGAC ACGAGCGCAA GCTTCTCTAT TAATCTGCTG CTCGTTTGCG    699
PUMY010-2    TATGTGGGAC ACGAGCGCAA GCTTCTCTAT TAATCTGCTG CTCGTTTGCG    699
PUMY010-3    TATGTGGGAC ACGAGCGCAA GCTTCTCTAT TAATCTGCTG CTCGTTTGCG    699
PUMY010-4    TATGTGGGAC ACGAGCGCAA GCTTCTCTAT TAATCTGCTG CTCGTTTGCG    700
PUMY010-5    TATGTGGGAC ACGAGCGCAA GCTTCTCTAT TAATCTGCTG CTCGTTTGCG    700
PUMY010-6    TATGTGGGAC ACGAGCGCAA GCTTCTCTAT TAATCTGCTG CTCGTTTGCG    699
PUMY010-7    TATGTGGGAC ACGAGCGCAA GCTTCTCTAT TAATCTGCTG CTCGTTTGCG    700

PUMY010-1    CGAGCGGCGG GGGTTAATAC TGTATTAGGT TTTACCAACT CGGTGTTGAT    749
PUMY010-2    CGAGCGGCGG GGGTTAATAC TGTATTAGGT TTTACCAACT CGGTGTTGAT    749
PUMY010-3    CGAGCGGCGG GGGTTAATAC TGTATTAGGT TTTACCAACT CGGTGTTGAT    749
PUMY010-4    CGAGCGGCGG GGGTTAATAC TGTATTAGGT TTTACCAACT CGGTGTTGAT    750
PUMY010-5    CGAGCGGCGG GGGTTAATAC TGTATTAGGT TTTACCAACT CGGTGTTGAT    750
PUMY010-6    CGAGCGGCGG GGGTTAATAC TGTATTAGGT TTTACCAACT CGGTGTTGAT    749
PUMY010-7    CGAGCGGCGG GGGTTAATAC TGTATTAGGT TTTACCAACT CGGTGTTGAT    750
                                      ●
PUMY010-1    CTAGGGAGGG ATAAGTGAGT GTTCTGTGCG TGCTGGGCAG ACAGACGTCT    799
PUMY010-2    CTAGGGAGGG ATAAGTGAGT GTTTTGTGCG TGCTGGGCAG ACAGACGTCT    799
PUMY010-3    CTAGGGAGGG ATAAGTGAGT GTTCTGTGCG TGCTGGGCAG ACAGACGTCT    799
PUMY010-4    CTAGGGAGGG ATAAGTGAGT GTTCTGTGCG TGCTGGGCAG ACAGACGTCT    800
PUMY010-5    CTAGGGAGGG ATAAGTGAGT GTTCTGTGCG TGCTGGGCAG ACAGACGTCT    800
PUMY010-6    CTAGGGAGGG ATAAGTGAGT GTTTTGTGCG TGCTGGGCAG ACAGACGTCT    799
PUMY010-7    CTAGGGAGGG ATAAGTGAGT GTTTTGTGCG TGCTGGGCAG ACAGACGTCT    800
                 25S nrDNA
PUMY010-1    TTAAGTTTGA CCTCAAATCA GGTAGGGTTA CCCGCTGAAC TTAA          843
PUMY010-2    TTAAGTTTGA CCTCAAATCA GGTAGGGTTA CCCGCTGAAC TTAA          843
PUMY010-3    TTAAGTTTGA CCTCAAATCA GGTAGGGTTA CCCGCTGAAC TTAA          843
PUMY010-4    TTAAGTTTGA CCTCAAATCA GGTAGGGTTA CCCGCTGAAC TTAA          844
PUMY010-5    TTAAGTTTGA CCTCAAATCA GGTAGGGTTA CCCGCTGAAC TTAA          844
PUMY010-6    TTAAGTTTGA CCTCAAATCA GGTAGGGTTA CCCGCTGAAC TTAA          843
PUMY010-7    TTAAGTTTGA CCTCAAATCA GGTAGGGTTA CCCGCTGAAC TTAA          844
```

Figure S2. *Cont.*

```
                18S nrDNA   ITS1
PUMY011-1       AAGGATCATT AAAGAAATTT AATTGATTTG TCTGAGCTCG GAGAGAGACA    50
PUMY011-2       AAGGATCATT AAAGAAATTT AATTGATTTG TCTGAGCTCG GAGAGAGACA    50
PUMY011-3       AAGGATCATT AAAGAAATTT AATTGATTTG TCTGAGCTCG GAGAGAGACA    50
                                                      ●
PUMY011-1       TCTCTGGGGA GGACCAGTGT AGACACTCAG GAGGCACCTA AAATATTTTC    100
PUMY011-2       TCTCTGGGGA GGACCAGTGT AGACACTCAG GAGGCTCCTA AAATATTTTC    100
PUMY011-3       TCTCTGGGGA GGACCAGTGT AGACACTCAG GAGGCACCTA AAATATTTTC    100

PUMY011-1       TCTGCTGTGA ATGCTATTTC TCCTGCCTGC GCTTAAGTGC GCGGTTGGTG    150
PUMY011-2       TCTGCTGTGA ATGCTATTTC TCCTGCCTGC GCTTAAGTGC GCGGTTGGTG    150
PUMY011-3       TCTGCTGTGA ATGCTATTTC TCCTGCCTGC GCTTAAGTGC GCGGTTGGTG    150

PUMY011-1       GGTGTTCTGC AGTGGGGGGA GGGAGCCGAC AAAGACCTGG GAGTGTGCGT    200
PUMY011-2       GGTGTTCTGC AGTGGGGGGA GGGAGCCGAC AAAGACCTGG GAGTGTGCGT    200
PUMY011-3       GGTGTTCTGC AGTGGGGGGA GGGAGCCGAC AAAGACCTGG GAGTGTGCGT    200

PUMY011-1       GGATCTCTCT ATTCCAAAGG AGGTGTTTTA TCACACGACT CGACACTTTC    250
PUMY011-2       GGATCTCTCT ATTCCAAAGG AGGTGTTTTA TCACACGACT CGACACTTTC    250
PUMY011-3       GGATCTCTCT ATTCCAAAGG AGGTGTTTTA TCACACGACT CGACACTTTC    250

PUMY011-1       TAATTACTAC ACACAGTGGA GTTTACTTTA CTACTATTCT TTTGTTCGTT    300
PUMY011-2       TAATTACTAC ACACAGTGGA GTTTACTTTA CTACTATTCT TTTGTTCGTT    300
PUMY011-3       TAATTACTAC ACACAGTGGA GTTTACTTTA CTACTATTCT TTTGTTCGTT    300

PUMY011-1       GGGGGAAAGC TCTCTTTCGG GAGGGAGTTC TCCCAGTGGA TGCAAACACA    350
PUMY011-2       GGGGGAAAGC TCTCTTTCGG GAGGGAGTTC TCCCAGTGGA TGCAAACACA    350
PUMY011-3       GGGGGAAAGC TCTCTTTCGG GAGGGAGTTC TCCCAGTGGA TGCAAACACA    350
                                 ●●●●   ●
PUMY011-1       AACAAATATT TTTTTATTTT AACTAATTCA GTCAACACAA GATTTCTTTT    400
PUMY011-2       AACAAATATT TTTTTA---- AAATAATTCA GTCAACACAA GATTTCTTTT    396
PUMY011-3       AACAAATATT TTTTTA---- AAATAATTCA GTCAACACAA GATTTCTTTT    396
                                 5.8S nrDNA
PUMY011-1       AGTAGAAAAC AACTTCAAAA CTTTCAACAA TGGATCTCTT GGTTCTCGCA    450
PUMY011-2       AGTAGAAAAC AACTTCAAAA CTTTCAACAA TGGATCTCTT GGTTCTCGCA    446
PUMY011-3       AGTAGAAAAC AACTTCAAAA CTTTCAACAA TGGATCTCTT GGTTCTCGCA    446

PUMY011-1       TCGATGAAGA ACGCAGCGAA ATGCGATACG TAATGTGAAT TGCAGAATTC    500
PUMY011-2       TCGATGAAGA ACGCAGCGAA ATGCGATACG TAATGTGAAT TGCAGAATTC    496
PUMY011-3       TCGATGAAGA ACGCAGCGAA ATGCGATACG TAATGTGAAT TGCAGAATTC    496

PUMY011-1       CGTGAATCAT CGAATCTTTG AACGCACATT GCGCCCTCTG GTATTCCGGG    550
PUMY011-2       CGTGAATCAT CGAATCTTTG AACGCACATT GCGCCCTCTG GTATTCCGGG    546
PUMY011-3       CGTGAATCAT CGAATCTTTG AACGCACATT GCGCCCTCTG GTATTCCGGG    546
                                           ITS2
PUMY011-1       GGGCATGCCT GTTTGAGCGT CATTTCCTTC TCAAACACGT TGTGTTTGGT    600
PUMY011-2       GGGCATGCCT GTTTGAGCGT CATTTCCTTC TCAAACACGT TGTGTTTGGT    596
PUMY011-3       GGGCATGCCT GTTTGAGCGT CATTTCCTTC TCAAACACGT TGTGTTTGGT    596

PUMY011-1       AGTGAGTGAT ACTCTCGTTT TTGAGTTAAC TTGAAATTGT AGGCCATATC    650
PUMY011-2       AGTGAGTGAT ACTCTCGTTT TTGAGTTAAC TTGAAATTGT AGGCCATATC    646
PUMY011-3       AGTGAGTGAT ACTCTCGTTT TTGAGTTAAC TTGAAATTGT AGGCCATATC    646

PUMY011-1       AGTATGTGGG ACACGAGCGC AAGCTTCTCT ATTAATCTGC TGCTCGTTTG    700
PUMY011-2       AGTATGTGGG ACACGAGCGC AAGCTTCTCT ATTAATCTGC TGCTCGTTTG    696
PUMY011-3       AGTATGTGGG ACACGAGCGC AAGCTTCTCT ATTAATCTGC TGCTCGTTTG    696

PUMY011-1       CGCGAGCGGC GGGGGTTAAT ACTGTATTAG GTTTTACCAA CTCGGTGTTG    750
PUMY011-2       CGCGAGCGGC GGGGGTTAAT ACTGTATTAG GTTTTACCAA CTCGGTGTTG    746
PUMY011-3       CGCGAGCGGC GGGGGTTAAT ACTGTATTAG GTTTTACCAA CTCGGTGTTG    746

PUMY011-1       ATCTAGGGAG GGATAAGTGA GTGTTTTGTG CGTGCTGGGC AGACAGACGT    800
PUMY011-2       ATCTAGGGAG GGATAAGTGA GTGTTTTGTG CGTGCTGGGC AGACAGACGT    796
PUMY011-3       ATCTAGGGAG GGATAAGTGA GTGTTTTGTG CGTGCTGGGC AGACAGACGT    796
                     5.8S nrDNA
PUMY011-1       CTTTAAGTTT GACCTCAAAT CAGGTAGGGT TACCCGCTGA ACTTAA        846
PUMY011-2       CTTTAAGTTT GACCTCAAAT CAGGTAGGGT TACCCGCTGA ACTTAA        842
PUMY011-3       CTTTAAGTTT GACCTCAAAT CAGGTAGGGT TACCCGCTGA ACTTAA        842
```

Figure S2. *Cont.*

```
                 18S nrDNA   ITS1
PUMY020-1        AAGGATCATT ACTGTGATTT AAACTTCTTT CTTACACCGC GTGAGCGCAC    50
PUMY020-2        AAGGATCATT ACTGTGATTT AAACTTCTTT CTTACACCGC GTGAGCGCAC    50
PUMY020-3        AAGGATCATT ACTGTGATTT AAACTTCTTT CTTACACCGC GTGAGCGCAC    50
PUMY020-4        AAGGATCATT ACTGTGATTT AAACTTCTTT CTTACACCGC GTGAGCGCAC    50

PUMY020-1        AACAACACCT AAACACGAAT AACCATGTCA CCCAGAGAAA ATCTCAAACG    100
PUMY020-2        AACAACACCT AAACACGAAT AACCATGTCA CCCAGAGAAA ATCTCAAACG    100
PUMY020-3        AACAACACCT AAACACGAAT AACCATGTCA CCCAGAGAAA ATCTCAAACG    100
PUMY020-4        AACAACACCT AAACACGAAT AACCATGTCA CCCAGAGAAA ATCTCAAACG    100
                         •   •         5.8S nrDNA
PUMY020-1        AGAAGAAA-A AGAAAAATAA AACTTTCAAC AACGGATCTC TTGGTTCTCG    149
PUMY020-2        AGAAGAAA-A AGAAAAATAA AACTTTCAAC AACGGATCTC TTGGTTCTCG    149
PUMY020-3        AGAAGAAAGA AAAAAAATAA AACTTTCAAC AACGGATCTC TTGGTTCTCG    150
PUMY020-4        AGAAGAAAGA AAAAAAATAA AACTTTCAAC AACGGATCTC TTGGTTCTCG    150

PUMY020-1        CATCGATGAA GAGCGCAGCG AAATGCGATA CCTAGTGTGA ATTGCAGCCA    199
PUMY020-2        CATCGATGAA GAGCGCAGCG AAATGCGATA CCTAGTGTGA ATTGCAGCCA    199
PUMY020-3        CATCGATGAA GAGCGCAGCG AAATGCGATA CCTAGTGTGA ATTGCAGCCA    200
PUMY020-4        CATCGATGAA GAGCGCAGCG AAATGCGATA CCTAGTGTGA ATTGCAGCCA    200

PUMY020-1        TCGTGAATCA TCGAGTTCTT GAACGCACAT TGCGCCCTCC GGCATTCCGG    249
PUMY020-2        TCGTGAATCA TCGAGTTCTT GAACGCACAT TGCGCCCTCC GGCATTCCGG    249
PUMY020-3        TCGTGAATCA TCGAGTTCTT GAACGCACAT TGCGCCCTCC GGCATTCCGG    250
PUMY020-4        TCGTGAATCA TCGAGTTCTT GAACGCACAT TGCGCCCTCC GGCATTCCGG    250
                                        ITS2
PUMY020-1        GGGGCATGCC TGTTTGAGCG TCGTTTCCTT CTTGCGCAAG CAGAGTTGGG    299
PUMY020-2        GGGGCATGCC TGTTTGAGCG TCGTTTCCTT CTTGCGCAAG CAGAGTTGGG    299
PUMY020-3        GGGGCATGCC TGTTTGAGCG TCGTTTCCTT CTTGCGCAAG CAGAGTTGGG    300
PUMY020-4        GGGGCATGCC TGTTTGAGCG TCGTTTCCTT CTTGCGCAAG CAGAGTTGGG    300

PUMY020-1        GTTGCCACGG CCCGTGCGGC CTGTGTGTGG CTCCCCCGAA ACGGAACGGC    349
PUMY020-2        GTTGCCACGG CCCGTGCGGC CTGTGTGTGG CTCCCCCGAA ACGGAACGGC    349
PUMY020-3        GTTGCCACGG CCCGTGCGGC CTGTGTGTGG CTCCCCCGAA ACGGAACGGC    350
PUMY020-4        GTTGCCACGG CCCGTGCGGC CTGTGTGTGG CTCCCCCGAA ACGGAACGGC    350

PUMY020-1        AGCGGGACTG AGCGAAGTAC ACAACACTCG CGCTTGGCCC GCCGAACTTT    399
PUMY020-2        AGCGGGACTG AGCGAAGTAC ACAACACTCG CGCTTGGCCC GCCGAACTTT    399
PUMY020-3        AGCGGGACTG AGCGAAGTAC ACAACACTCG CGCTTGGCCC GCCGAACTTT    400
PUMY020-4        AGCGGGACTG AGCGAAGTAC ACAACACTCG CGCTTGGCCC GCCGAACTTT    400
                       •          25S nrDNA
PUMY020-1        TTTTTTAAAT CTAAGCTCGA CCTCAAATCA GGTAGGAATA CCCGCTGAAC    449
PUMY020-2        TTTTTT-AAT CTAAGCTCGA CCTCAAATCA GGTAGGAATA CCCGCTGAAC    448
PUMY020-3        TTTTTTAAAT CTAAGCTCGA CCTCAAATCA GGTAGGAATA CCCGCTGAAC    450
PUMY020-4        TTTTTT-AAT CTAAGCTCGA CCTCAAATCA GGTAGGAATA CCCGCTGAAC    449

PUMY020-1        TTAA                                                      453
PUMY020-2        TTAA                                                      452
PUMY020-3        TTAA                                                      454
PUMY020-4        TTAA                                                      453
```

Figure S2. *Cont.*

```
              18S nrDNA   ITS1
PUMY021-1    AAGGATCATT ACTGTGATTT AAACTTCTTT CTTACACCGC GTGAGCGCAC    50
PUMY021-2    AAGGATCATT ACTGTGATTT AAACTTCTTT CTTACACCGC GTGAGCGCAC    50
PUMY021-3    AAGGATCATT ACTGTGATTT AAACTTCTTT CTTACACCGC GTGAGCGCAC    50
PUMY021-4    AAGGATCATT ACTGTGATTT AAACTTCTTT CTTACACCGC GTGAGCGCAC    50
PUMY021-5    AAGGATCATT ACTGTGATTT AAACTTCTTT CTTACACCGC GTGAGCGCAC    50

PUMY021-1    AACAACACCT AAACACGAAT AACCATGTCA CCCAGAGAAA ATCTCAAACG    100
PUMY021-2    AACAACACCT AAACACGAAT AACCATGTCA CCCAGAGAAA ATCTCAAACG    100
PUMY021-3    AACAACACCT AAACACGAAT AACCATGTCA CCCAGAGAAA ATCTCAAACG    100
PUMY021-4    AACAACACCT AAACACGAAT AACCATGTCA CCCAGAGAAA ATCTCAAACG    100
PUMY021-5    AACAACACCT AAACACGAAT AACCATGTCA CCCAGAGAAA ATCTCAAACG    100
                     •   •           5.8S nrDNA
PUMY021-1    AGAAGAAA-A AGAAAAATAA AACTTTCAAC AACGGATCTC TTGGTTCTCG    149
PUMY021-2    AGAAGAAA-A AGAAAAATAA AACTTTCAAC AACGGATCTC TTGGTTCTCG    149
PUMY021-3    AGAAGAAAGA AAAAAAATAA AACTTTCAAC AACGGATCTC TTGGTTCTCG    150
PUMY021-4    AGAAGAAAGA AAAAAAATAA AACTTTCAAC AACGGATCTC TTGGTTCTCG    150
PUMY021-5    AGAAGAAAGA AAAAAAATAA AACTTTCAAC AACGGATCTC TTGGTTCTCG    150

PUMY021-1    CATCGATGAA GAGCGCAGCG AAATGCGATA CCTAGTGTGA ATTGCAGCCA    199
PUMY021-2    CATCGATGAA GAGCGCAGCG AAATGCGATA CCTAGTGTGA ATTGCAGCCA    199
PUMY021-3    CATCGATGAA GAGCGCAGCG AAATGCGATA CCTAGTGTGA ATTGCAGCCA    200
PUMY021-4    CATCGATGAA GAGCGCAGCG AAATGCGATA CCTAGTGTGA ATTGCAGCCA    200
PUMY021-5    CATCGATGAA GAGCGCAGCG AAATGCGATA CCTAGTGTGA ATTGCAGCCA    200

PUMY021-1    TCGTGAATCA TCGAGTTCTT GAACGCACAT TGCGCCCTCC GGCATTCCGG    249
PUMY021-2    TCGTGAATCA TCGAGTTCTT GAACGCACAT TGCGCCCTCC GGCATTCCGG    249
PUMY021-3    TCGTGAATCA TCGAGTTCTT GAACGCACAT TGCGCCCTCC GGCATTCCGG    250
PUMY021-4    TCGTGAATCA TCGAGTTCTT GAACGCACAT TGCGCCCTCC GGCATTCCGG    250
PUMY021-5    TCGTGAATCA TCGAGTTCTT GAACGCACAT TGCGCCCTCC GGCATTCCGG    250
                                    ITS2
PUMY021-1    GGGGCATGCC TGTTTGAGCG TCGTTTCCTT CTTGCGCAAG CAGAGTTGGG    299
PUMY021-2    GGGGCATGCC TGTTTGAGCG TCGTTTCCTT CTTGCGCAAG CAGAGTTGGG    299
PUMY021-3    GGGGCATGCC TGTTTGAGCG TCGTTTCCTT CTTGCGCAAG CAGAGTTGGG    300
PUMY021-4    GGGGCATGCC TGTTTGAGCG TCGTTTCCTT CTTGCGCAAG CAGAGTTGGG    300
PUMY021-5    GGGGCATGCC TGTTTGAGCG TCGTTTCCTT CTTGCGCAAG CAGAGTTGGG    300

PUMY021-1    GTTGCCACGG CCCGTGCGGC CTGTGTGTGG CTCCCCCGAA ACGGAACGGC    349
PUMY021-2    GTTGCCACGG CCCGTGCGGC CTGTGTGTGG CTCCCCCGAA ACGGAACGGC    349
PUMY021-3    GTTGCCACGG CCCGTGCGGC CTGTGTGTGG CTCCCCCGAA ACGGAACGGC    350
PUMY021-4    GTTGCCACGG CCCGTGCGGC CTGTGTGTGG CTCCCCCGAA ACGGAACGGC    350
PUMY021-5    GTTGCCACGG CCCGTGCGGC CTGTGTGTGG CTCCCCCGAA ACGGAACGGC    350

PUMY021-1    AGCGGGACTG AGCGAAGTAC ACAACACTCG CGCTTGGCCC GCCGAACTTT    399
PUMY021-2    AGCGGGACTG AGCGAAGTAC ACAACACTCG CGCTTGGCCC GCCGAACTTT    399
PUMY021-3    AGCGGGACTG AGCGAAGTAC ACAACACTCG CGCTTGGCCC GCCGAACTTT    400
PUMY021-4    AGCGGGACTG AGCGAAGTAC ACAACACTCG CGCTTGGCCC GCCGAACTTT    400
PUMY021-5    AGCGGGACTG AGCGAAGTAC ACAACACTCG CGCTTGGCCC GCCGAACTTT    400
                   •          25S nrDNA
PUMY021-1    TTTTTTAAAT CTAAGCTCGA CCTCAAATCA GGTAGGAATA CCCGCTGAAC    449
PUMY021-2    TTTTTT-AAT CTAAGCTCGA CCTCAAATCA GGTAGGAATA CCCGCTGAAC    448
PUMY021-3    TTTTTTAAAT CTAAGCTCGA CCTCAAATCA GGTAGGAATA CCCGCTGAAC    450
PUMY021-4    TTTTTT-AAT CTAAGCTCGA CCTCAAATCA GGTAGGAATA CCCGCTGAAC    449
PUMY021-5    TTTTTTTAAT CTAAGCTCGA CCTCAAATCA GGTAGGAATA CCCGCTGAAC    450

PUMY021-1    TTAA                                                      453
PUMY021-2    TTAA                                                      452
PUMY021-3    TTAA                                                      454
PUMY021-4    TTAA                                                      453
PUMY021-5    TTAA                                                      454
```

Figure S2. *Cont.*

```
                 18S nrDNA   ITS1
PUMY040-1        AAGGATCATT ACTGATTTGC TTAATTGCAC CACATGTGTT TTTTATTGAA    50
PUMY040-2        AAGGATCATT ACTGATTTGC TTAATTGCAC CACATGTGTT TTTTATTGAA    50
PUMY040-3        AAGGATCATT ACTGATTTGC TTAATTGCAC CACATGTGTT TTTTATTGAA    50

PUMY040-1        CAAATTTCTT TGGTGGCGGG AGCAATCCTA CCGCCAGAGG TTATAACTAA    100
PUMY040-2        CAAATTTCTT TGGTGGCGGG AGCAATCCTA CCGCCAGAGG TTATAACTAA    100
PUMY040-3        CAAATTTCTT TGGTGGCGGG AGCAATCCTA CCGCCAGAGG TTATAACTAA    100

PUMY040-1        ACCAAACTTT TTATTTACAG TCAAACTTGA TTTATTATTA CAATAGTCAA    150
PUMY040-2        ACCAAACTTT TTATTTACAG TCAAACTTGA TTTATTATTA CAATAGTCAA    150
PUMY040-3        ACCAAACTTT TTATTTACAG TCAAACTTGA TTTATTATTA CAATAGTCAA    150
                 5.8S nrDNA
PUMY040-1        AACTTTCAAC AACGGATCTC TTGGTTCTCG CATCGATGAA GAACGCAGCG    200
PUMY040-2        AACTTTCAAC AACGGATCTC TTGGTTCTCG CATCGATGAA GAACGCAGCG    200
PUMY040-3        AACTTTCAAC AACGGATCTC TTGGTTCTCG CATCGATGAA GAACGCAGCG    200

PUMY040-1        AAATGCGATA CGTAATATGA ATTGCAGATA TTCGTGAATC ATCGAATCTT    250
PUMY040-2        AAATGCGATA CGTAATATGA ATTGCAGATA TTCGTGAATC ATCGAATCTT    250
PUMY040-3        AAATGCGATA CGTAATATGA ATTGCAGATA TTCGTGAATC ATCGAATCTT    250

PUMY040-1        TGAACGCACA TTGCGCCCTT TGGTATTCCA AAGGGCATGC CTGTTTGAGC    300
PUMY040-2        TGAACGCACA TTGCGCCCTT TGGTATTCCA AAGGGCATGC CTGTTTGAGC    300
PUMY040-3        TGAACGCACA TTGCGCCCTT TGGTATTCCA AAGGGCATGC CTGTTTGAGC    300
                             ITS2
PUMY040-1        GTCATTTCTC CCTCAAACCC CCGGGTTTGG TGTTGAGCAA TACGCTAGGT    350
PUMY040-2        GTCATTTCTC CCTCAAACCC CCGGGTTTGG TGTTGAGCAA TACGCTAGGT    350
PUMY040-3        GTCATTTCTC CCTCAAACCC CCGGGTTTGG TGTTGAGCAA TACGCTAGGT    350
                                   ●
PUMY040-1        TTGTTTGAAA GAATTT-ACG TGGAAACTTA TTTTAAGCGA CTTAGGTTTA    399
PUMY040-2        TTGTTTGAAA GAATTT-ACG TGGAAACTTA TTTTAAGCGA CTTAGGTTTA    399
PUMY040-3        TTGTTTGAAA GAATTTAACG TGGAAACTTA TTTTAAGCGA CTTAGGTTTA    400
                    ●                                        25S nrDNA
PUMY040-1        TCCAAAAACG CTTATTTTGC TAGTGGCCAC CACAATTTAT TTCATAACTT    449
PUMY040-2        TCC-AAAACG CTTATTTTGC TAGTGGCCAC CACAATTTAT TTCATAACTT    448
PUMY040-3        TCCAAAAACG CTTATTTTGC TAGTGGCCAC CACAATTTAT TTCATAACTT    450

PUMY040-1        TGACCTCAAA TCAGGTAGGA CTACCCGCTG AACTTAA                  486
PUMY040-2        TGACCTCAAA TCAGGTAGGA CTACCCGCTG AACTTAA                  485
PUMY040-3        TGACCTCAAA TCAGGTAGGA CTACCCGCTG AACTTAA                  487
```

Figure S2. *Cont.*

```
                18S nrDNA   ITS1                             •
PUMY065-1     AAGGATCATT AAAGAAATTT AATAATTTTG AAAATGGATT TTTTTTGTTT    50
PUMY065-2     AAGGATCATT AAAGAAATTT AATAATTTTG AAAATGGA-T TTTTTTGTTT    49

PUMY065-1     TGGCAAGAGC ATGAGAGCTT TTACTGGGCA AGAAGACAAG AGATGGAGAG    100
PUMY065-2     TGGCAAGAGC ATGAGAGCTT TTACTGGGCA AGAAGACAAG AGATGGAGAG    99

PUMY065-1     TCCAGCCGGG CCTGCGCTTA AGTGCGCGGT CTTGCTAGGC TTGTAAGTTT    150
PUMY065-2     TCCAGCCGGG CCTGCGCTTA AGTGCGCGGT CTTGCTAGGC TTGTAAGTTT    149

PUMY065-1     CTTTCTTGCT ATTCCAAACG GTGAGAGATT TCTGTGCTTT TGTTATAGGA    200
PUMY065-2     CTTTCTTGCT ATTCCAAACG GTGAGAGATT TCTGTGCTTT TGTTATAGGA    199

PUMY065-1     CAATTAAAAC CGTTTCAATA CAACACACTG TGGAGTTTTC ATATCTTTGC    250
PUMY065-2     CAATTAAAAC CGTTTCAATA CAACACACTG TGGAGTTTTC ATATCTTTGC    249

PUMY065-1     AACTTTTTCT TTGGGCATTC GAGCAATCGG GGCCCAGAGG TAACAAACAC    300
PUMY065-2     AACTTTTTCT TTGGGCATTC GAGCAATCGG GGCCCAGAGG TAACAAACAC    299

PUMY065-1     AAACAATTTT ATTTATTCAT TAAATTTTTG TCAAAAACAA GAATTTTCGT    350
PUMY065-2     AAACAATTTT ATTTATTCAT TAAATTTTTG TCAAAAACAA GAATTTTCGT    349
                                         5.8S nrDNA
PUMY065-1     AACTGGAAAT TTTAAAATAT TAAAAACTTT CAACAACGGA TCTCTTGGTT    400
PUMY065-2     AACTGGAAAT TTTAAAATAT TAAAAACTTT CAACAACGGA TCTCTTGGTT    399

PUMY065-1     CTCGCATCGA TGAAGAACGC AGCGAAATGC GATACGTAAT GTGAATTGCA    450
PUMY065-2     CTCGCATCGA TGAAGAACGC AGCGAAATGC GATACGTAAT GTGAATTGCA    449

PUMY065-1     GAATTCCGTG AATCATCGAA TCTTTGAACG CACATTGCGC CCCTTGGTAT    500
PUMY065-2     GAATTCCGTG AATCATCGAA TCTTTGAACG CACATTGCGC CCCTTGGTAT    499
                                               ITS2
PUMY065-1     TCCAGGGGGC ATGCCTGTTT GAGCGTCATT TCCTTCTCAA ACATTCTGTT    550
PUMY065-2     TCCAGGGGGC ATGCCTGTTT GAGCGTCATT TCCTTCTCAA ACATTCTGTT    549

PUMY065-1     TGGTAGTGAG TGATACTCTT TGGAGTTAAC TTGAAATTGC TGGCCTTTTC    600
PUMY065-2     TGGTAGTGAG TGATACTCTT TGGAGTTAAC TTGAAATTGC TGGCCTTTTC    599

PUMY065-1     ATTGGATGTT TTTTTTCCAA AGAGAGGTTT CTCTGCGTGC TTGAGGTATA    650
PUMY065-2     ATTGGATGTT TTTTTTCCAA AGAGAGGTTT CTCTGCGTGC TTGAGGTATA    649

PUMY065-1     ATGCAAGTAC GGTCGTTTTA GGTTTTACCA ACTGCGGCTA ATCTTTTTTA    700
PUMY065-2     ATGCAAGTAC GGTCGTTTTA GGTTTTACCA ACTGCGGCTA ATCTTTTTTA    699

PUMY065-1     TACTGAGCGT ATTGGAACGT TATCGATAAG AAGAGAGCGT CTAGGCGAAC    750
PUMY065-2     TACTGAGCGT ATTGGAACGT TATCGATAAG AAGAGAGCGT CTAGGCGAAC    749
                                  28S nrDNA
PUMY065-1     AATGTTCTTA AAGTTTGACC TCAAATCAGG TAGGAGTACC CGCTGAACTT    800
PUMY065-2     AATGTTCTTA AAGTTTGACC TCAAATCAGG TAGGAGTACC CGCTGAACTT    799

PUMY065-1     AA                                                        802
PUMY065-2     AA                                                        801
```

Figure S2. Multiple alignments of ITS sequences of the six yeast strains. The internal transcribed spacer regions ITS1 and ITS2 are highlighted in gray, and the intra-genomic polymorphic sites are indicated by solid black dots above the corresponding nucleotides.
